# The Investigation of Electrochemistry Behaviors of Tyrosinase Based on Directly-Electrodeposited Grapheneon Choline-Gold Nanoparticles

**DOI:** 10.3390/molecules22071047

**Published:** 2017-06-23

**Authors:** Yaping He, Xiaohui Yang, Quan Han, Jianbin Zheng

**Affiliations:** 1 School of Chemical Engineering, Xi’an University, Xi’an 710065, China; yangxh1127@aliyun.com; 2 Institute of Analytical Science/Shaanxi Provincial Key Laboratory of Electroanalytical Chemistry, Northwest University, Xi’an 710069, China; zhengjb@nwu.edu.cn

**Keywords:** electrochemistry, tyrosinase, graphene, choline-gold nanoparticals, catechol

## Abstract

A novel catechol (CA) biosensor was developed by embedding tyrosinase (Tyr) onto in situ electrochemical reduction graphene (EGR) on choline-functionalized gold nanoparticle (AuNPs-Ch) film. The results of UV-Vis spectra indicated that Tyr retained its original structure in the film, and an electrochemical investigation of the biosensor showed a pair of well-defined, quasi-reversible redox peaks with *E*_pa_ = −0.0744 V and *E*_pc_ = −0.114 V (vs. SCE) in 0.1 M, pH 7.0 sodium phosphate-buffered saline at a scan rate of 100 mV/s. The transfer rate constant *k_s_* is 0.66 s^−1^. The Tyr-EGR/AuNPs-Ch showed a good electrochemical catalytic response for the reduction of CA, with the linear range from 0.2 to 270 μM and a detection limit of 0.1 μM (S/N = 3). The apparent Michaelis-Menten constant was estimated to be 109 μM.

## 1. Introduction

Phenolic compounds are a very important and widespread class of substances [1]. However, they are toxic and injure mammals, fishes and other aquatic organisms [2]. Due to their toxicity and persistency in the environment, they are on the priority pollutants list of the European Community and the Environmental Protection Agency of the United States [3]. The determination of phenols is of paramount importance in environmental analysis, and medical and food quality characterization. Spectrophotometry and chromatography are commonly-useful methods for the determination of phenols [4]. These techniques have a number of disadvantages, such as expense, time-consumption, and difficult in situ application [3], which limit their applications to laboratory settings and rapid analyses under field conditions. To overcome the defects mentioned above, a simple and effective analytical method for the determination of phenols is needed. An electrochemical method has been considered as the best choice for in situ monitoring of phenolic compounds by virtue of its high sensitivity, simple instrumentation, low production cost, and promising response speed [5]. The high applied voltage needed in direct electrochemistry of phenols [6] is also followed by an increase of the background current and noise level. Furthermore, direct electrochemical oxidation of phenols is coupled with fouling reactions [7]. Enzyme-based amperometric biosensors for the determination of phenols have been experimentally demonstrated as an efficient route to solve the obstacles mentioned above. Electrochemical biosensors based on enzymes have been used for the determination of phenolic compounds in vivo and in vitro with high selectivity, sensitivity, and rapid analysis rates in various biological species [8]. Tyrosinase (Tyr), known as polyphenol oxidase, is a copper monooxygenase that catalyzes the oxidation of phenolic compounds to their corresponding *o*-quinines [9]. It belongs to the Type 3 copper proteins family, a single copper center being responsible for the oxidation of phenolic substrates with oxygen as the co-substrate [10], and allows convenient low-potential detection of phenolic analytes [11]. Electrochemical biosensors based on Tyr are simple and convenient tools for phenol assays due to their high sensitivity, effectiveness, and simplicity [12]. Many nanomaterials, such as carbon-coated nickel nanoparticles [13], polyaniline (PANI) [14], hydroxyapatite [5], Fe_3_O_4_ nanoparticles [4], ZnO nanorod microarrays [15], and sonogel-carbon [16], have been used to construct Tyr biosensors for the detection of catechol (CA). It shows advantages of good reliability, fast response, inexpensive instrumentation, low energy consumption, simple operation, time savings, and high sensitivity [17]. Unfortunately, redox peaks, corresponding to the T3 site, could not be seen on cyclic voltammetry (CV) scans performed under anaerobic conditions [10]. Thus, the direct electrochemistry of Tyr is commonly difficult to be observed. There are only a few studies that described the direct electrochemistry of Tyr based on Woodward’s Reagent K [18], single-walled carbon nanotubes [11,19], gold nanoparticles (AuNPs) [20], and nickel oxide nanoparticles (NiONPs) [21].

Carbon can act as a primary electron donor to native electroactive sites of enzymes [16]. As the “shining star” in the carbon nanomaterial family, graphene (GR) has attracted more and more attention in the preparation of sensors and biosensors [22,23,24] due to their large surface area, extraordinary electronic transport property, high electrocatalytic activity, good mechanical strength, high thermal conductivity, and high mobility of charge carriers [25,26,27]. Our group has successfully achieved the direct chemistry of hemoglobin and glucose oxidase with GR-based materials [28,29]. Electrochemical methods are one of the most promising environmentally friendly (without using any toxic solvents) methods to obtain GR via graphene oxide (GO) and it is simple, fast, cost-effective, and it is more suitable for preparing less defective graphene sheets [30,31,32]. Sensors for the determination of ascorbic acid, hydrazine hydrate, and glucose have been fabricated by us through the direct electrodepositing GR on the surface of the bare electrode [33,34,35]. GR functionalized with biocompatible materials [36] makes it water-soluble and biocompatible, which can act as a novel material, promising for biological applications [37]. Biocompatible GR as a sensor platform not only presents an abundant domain for bimolecular binding for signal amplification, but also accelerates electron-transfer kinetics in electrochemical detection [38].

AuNPs possess distinct physical and chemical attributes that make them excellent scaffolds for the fabrication of novel chemical and biological sensors [39]. Electrochemical studies have revealed that AuNPs can enhance electrode conductivity, facilitating the electron transfer and improving the detection limit for biomolecules [40]. Self-assembled monolayers of AuNPs attached to alkane or aromaticthiols terminated with functional groups -SH or -NH_2_ are useful for the preparation of AuNP-modified electrodes. AuNPs have been successfully used for bioanalytical applications because of their low cytotoxicity, high affinity with molecules of thiol/amine-containing groups, and suitable platforms for surface immobilization of a wide range of biomolecules [41]. Undoubtedly, the merits of GR and AuNPs contribute to the high sensitivity of biosensors [29]. Graphene-AuNPs composites have been well prepared and used as the electro–catalysts for biosensingof glucose, oxygen, and uric acid [42].

Under the condition of electrochemical oxidation, the hydroxyl of choline (Ch) reacted with the carboxyl on the surface of a glassy carbon electrode (GCE) to fabricate a –C–O–C– bond, which was covalently grafted on the surface of GCE [43]. The quaternary ammonium -N^+^(CH_3_)_3_ functional groups of the choline modified layer had a positive charge, which may interact with the negatively-charged nanobiocomposite, such as AuNPs [44]. The choline layer could not only attach Au colloids, but also promoted the direct electron transfer of proteins [43].

As mentioned above, a biocompatible GR film can be easily fabricated with Ch-AuNPs as the linker to bare GCE, and acted as the attachment sites for the direct electrodeposition of graphene (EGR). The aim of the present work is to fabricate an in situ EGR-AuNPs-Chbiocompatible film and immobilize Tyr onto it. The electrochemical behavior of Tyr and its electrocatalysis towards catechol (CA)in the proposed biosensor is further investigated.

## 2. Experimental

### 2.1. Materials andApparatus

Tyr from mushroom (3610 Umg^−1^), Nafion (5% *m*/*v* in methanol), and chloroauricacidtetrahydrate (HAuCl_4_·4H_2_O) were purchased from Sigma (Los Angeles, CA, USA). Choline chloride [HOC_2_H_4_N(CH_3_)_3_Cl] (ChCl) was purchased from Sinopharm Chemical Reagent Co., Ltd. (Shanghai, China), Ethylene glycol (EG) was obtained from Tianjin TianLi Chemical Reagent Ltd. (Tianjin, China). High-purity graphite powder was purchased from Shanghai Carbon Plant (Shanghai, China). CA was purchased from Tianjin Kemiou Chemical Reagent Co., Ltd. (Tianjin, China). All other chemicals were of analytical reagent grade and doubly-distilled water was used in all the experiments. A 0.1 M pH 7.0 sodium phosphate-buffered saline (PBS) was used in all electrochemical studies.

All electrochemical experiments were carried out on a CHI 660D electrochemical workstation (Shanghai CH Instrument Co., Ltd., Shanghai, China) using a three-electrode system. The working electrode was GCE or modified GCE. GCE of 3-mm diameter, before use, was first polished to a mirror-like surface with 1.0, 0.3 and 0.05 μm Al_2_O_3_ slurry on a polish cloth, and rinsed with double-distilled water, then sonicated in ethanol and double-distilled water for 5 min, respectively. Then, the electrode was allowed to dry under nitrogen. A saturated calomel electrode (SCE) and a platinum electrode served as reference and counter electrodes, respectively. UV-Vis spectra were obtained on a UV-Vis spectrophotometer attached to an Elx800 absorbance microplate reader (BioTek, Winooski, VT, USA). Herein, ITO (Hebei LingxianGaoke Co., Ltd., Shi Jia Zhuang, China) was used as the substrate for the investigation of the morphology. Before use, it was cleaned by sonication sequentially for 20 min in acetone, 10% KOH in ethanol, and distilled water. Scanning electron microscopic (SEM) measurements were carried out on a JSM-6700F microscope (Japan Electron Company, Tokio, Japan) at 15 kV. All of the electrochemical experiments were conducted at room temperature (25 ± 2 °C).

### 2.2. Preparation and Modification of Nafion/Tyr/EGR-AuNPs-Ch/GCE

EG and ChCl (2:1) were gentle heated by continuous stirring until a homogeneous solution formed. Five-hundred microliters (500 μL) of HAuCl_4_ solution (*w*/*w*, 1%) was added into 50 mL of the above solution. Then, 5 mL of sodium borohydride solution (*w*/*w*, 1%) was slowly added, while stirring vigorously. The wine red solution was kept stirring at 50 °C for another 30 min. Then, it was allowed it to cool and AuNPs-Ch was prepared and stored at 4 °C for use. 

GO was synthesized from graphite powder by a modified Hummers method [45]. The fabrication process of the Nafion/Tyr/EGR-AuNPs-Ch/GCE was as follows: 10 μL of AuNPs-Ch homogeneous solution was casted onto the surface of the GCE by using a syringe to prepare AuNPs-Ch/GCE. The modified electrode dried at room temperature later. The preparation of electrolyte solution: 1 mg/mL GO was dispersed in 1/15 M, pH 9.18 PBS, and mixed via ultrasonication for several minutes to form a homogeneous solution. Prior to experiments, the solution was deoxygenated with high-purity nitrogen gas. The electrochemical deposition of EGR on the AuNPs-Ch/GCE was performed in the above eletrolyte solution from −1.5 to 0.5 V for 10 cycles at a scan rate of 10 mV/s. The treated substrate electrode was washed with distilled water.Ten microliters (10 μL) of Tyr (5 mg/mL in pH 7.0 PBS) was cast onto the electrode surface by using a syringe to prepare Tyr/EGR-AuNPs-Ch/GCE. Then, 3 μL of 0.5 wt % Nafion was dropped on it. Dried at room temperature later, the sensor was stored at 4 °C when not in use.

## 3. Results and Discussion

### 3.1. Fabrication and Characterization of the Nafion/Tyr/EGR-AuNPs-Ch/GCE

**Scheme 1:**
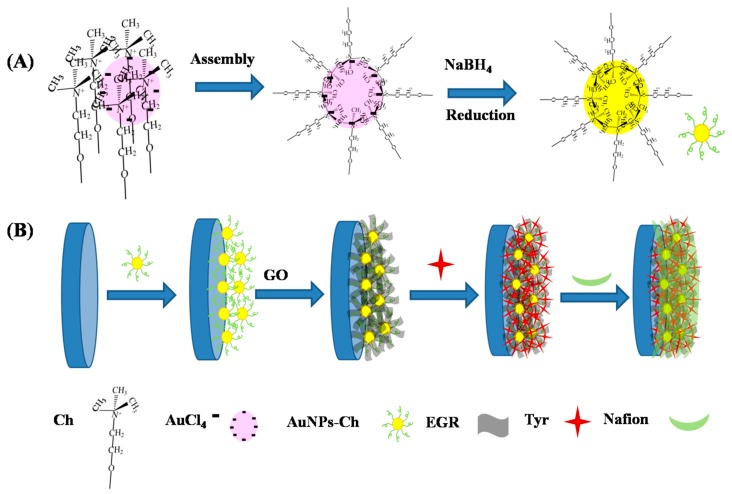
The fabrication strategy of AuNPs-Ch (**A**); and schematics of the fabrication process of the Nafion/Tyr/EGR-AuNPs-Ch/GCE (**B**).

As shown Scheme 1A, electrostatic assembly of the positively-charged -N^+^(CH_3_)_3_ polar head group of Ch with AuCl_4_^−^. The surface modification of gold nanospheres with choline was prepared by the reduction process, and the superficial Ch has hydroxy groups, which could be covalently bound to the edge plane sites of the carbon surface through the oxygen atom [46]. Thus, a –C–O–C– bond was formed, which efficiently immobilized AuNPs-Ch onto the bare GCE. AuNPs-Ch was fixed on the GCE, as shown in Scheme 1B. The presence of oxygen-containing groups [27] controlling GO exists as a planar sheet that can be inlaid into AuNPs-Ch to form large-scale two-dimensional arrays. The further in situelectrochemically-synthesized GR nanosheets enhanced the attachment of AuNPs-Ch with the electrode surface, which could form a stable EGR-AuNPs-Ch composite. Additionally, the high surface area of GR was helpful for immobilizing more proteins or enzymes and the nanocomposite film could provide a microenvironment for proteins or enzymes to retain their native structure and activity, and to achieve a reversible direct electron transfer reaction at the electrode surface [47]. Tyr anchored onto EGR and attached firmly. The addition of Nafion as an immobilization matrix to entrap enzymes and proteins effectively prevented the leakage of Tyr at the outermost point.

**Figure 1:**
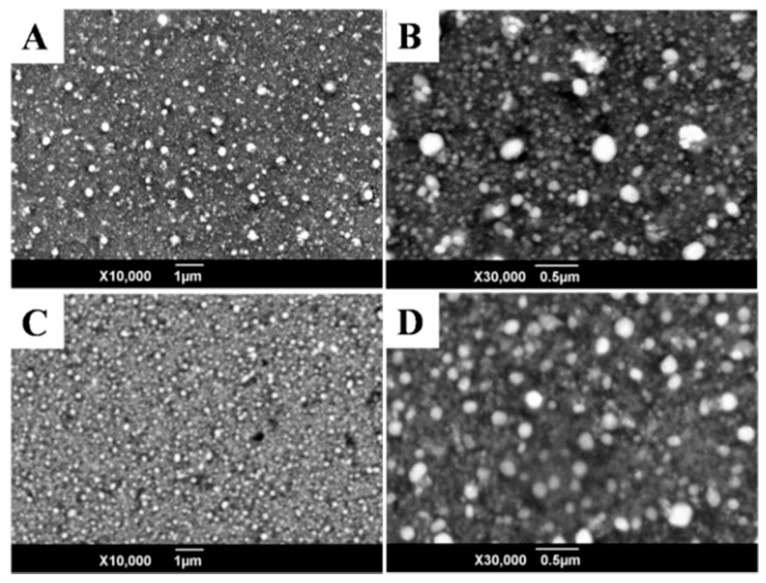
Scanning electron microscope (SEM) images of E-AuNPs-Chat low magnification (**A**) and high magnification (**B**); SEM images of EGR-AuNPs-Chat low magnification (**C**) and high magnification (**D**).

As shown in Figure 1A, with the electrochemical process from −1.5 to 0.5 V for 10 cycles at a scan rate of 10 mV/s, the AuNPs-Chnanospheres with diameters of 70 to 150 nm were heterogeneously scatteredon the surface of the sample because of agglomerate (as shown in Figure 1B). The much smaller homogeneously-scattered AuNPs had meandiameters of 30 to 50 in matrices. However, EGR-AuNPs-Ch showed to be much more uniform with evenly-dispersed nanospheres (Figure 1C). The EGR-AuNPs-Chshowed meandiameters of 50 to 120 nm (as shown in Figure 1D). EGR intercalated the structure of AuNPs-Ch, which restrained the GR layer overlap and AuNPs agglomeration, creating a favorable foundation for Tyr.

**Figure 2:**
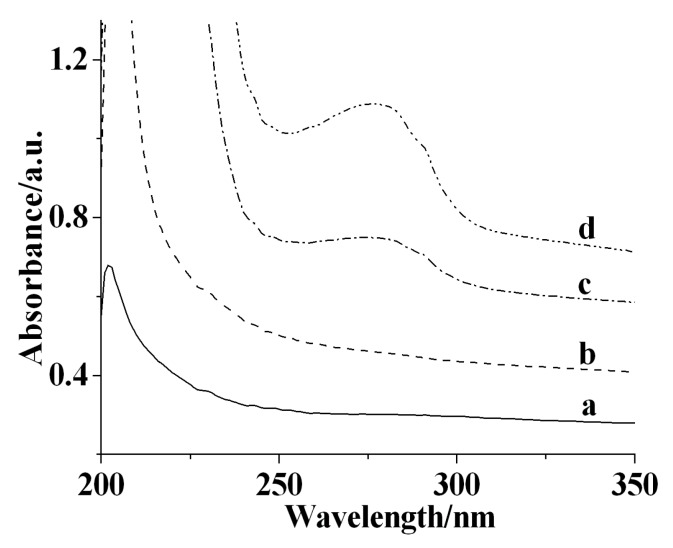
UV-Vis absorption spectra of AuNPs-Ch in PBS (a), EGR-AuNPs-Ch in PBS (b) in the solution of the Tyr/EGR-AuNPs-Ch mixture (c) and 0.3 mg/mL Tyr in PBS (d). The path length is 0.05 cm.

The UV-Vis Soretabsorption band of Tyr may provide the information on the conformational integrity of the protein and the possible denaturation or conformational change of the active center region. At a wavelength range from 200 to 350 nm, AuNPs-Ch (Figure 2, curve a) and EGR-AuNPs-Ch had no Soret band, indicating there is no interference of Tyr. Tyr dissolved in PBS had the Soret band at 279.8 nm (Figure 2, curve d), while the Soret band of the Tyr/EGR-AuNPs-Ch appeared at 280.3 nm (Figure 2, curve d). The difference of Tyr and the Tyr/EGR-AuNPs-Ch was less than 1 nm, indicating that Tyr in the Tyr/EGR-AuNPs-Ch had a microenvironment similar to the native state of Tyr in PBS.

**Figure 3:**
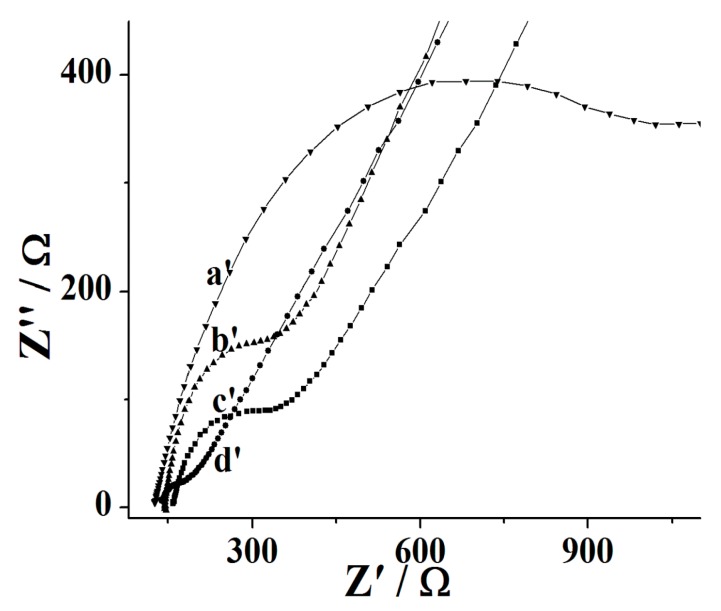
EIS plots for the bare GCE (a′), theAuNPs-Ch/GCE (b′), the Nafion/Tyr/EGR-AuNPs-Ch/GCE (c′) and the EGR-AuNPs-Ch/GCE (d′) in a solution of 5 mM [Fe(CN)_6_]^3−/4−^ + 0.1 M KCl as the supporting electrolyte. The frequencies were swept from 10^5^ to 10^−2^ Hz.

Electrochemical impedance spectroscopy (EIS) is an effective method for probing the features of surface modified electrode and can provide information on the impedance changes accompanying the stepwise electrode modification process. As shown in Figure 3, the semicircle of the AuNPs-Ch/GCE (curve b′) and the EGR-AuNPs-Ch/GCE (curve d′) were obviously smaller than that of the bare GCE (curve a′). The addition of Tyr blocked the electron transfer on the surface of GCE, resulted the increase of the impedance (curve c′), which was in good agreement with the results of the CVs.

### 3.2. Direct Electrochemistry of Tyr on the Nafion/Tyr/EGR-AuNPs-Ch/GCE

**Figure 4:**
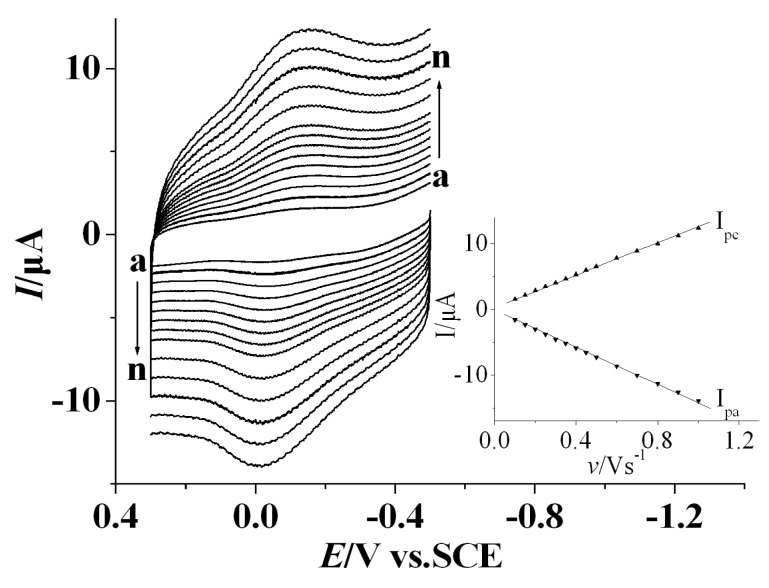
CVs of the Nafion/Tyr/EGR-AuNPs-Ch/GCE in N_2_-saturated PBS with different scan rates (from a to n: 100, 150, 200, 250, 300, 350, 400, 450, 500, 600, 700, 800, 900 and 1000 mV/s). Inset: the relationship between cathodic and anodic peak current with scan rate *v*.

The direct electrochemisty of Tyron the Nafion/Tyr/EGR-AuNPs-Ch/GCE was studied by cyclic voltammetry (CV). Cyclic voltammograms (CVs) of Tyrwith different scan rates were shown in Figure 4. The Nafion/Tyr/EGR-AuNPs-Ch/GCE showed a pair of well-defined, quasi-reversible redox peaks with *E_pa_* = −0.0744 V and *E_p_*_c_ = −0.114 V (vs. SCE) in PBS (0.1 M, pH 7.0) with the formal potential *E*^0^*′* = −0.0942 V. The value of *E*^0^*′* corresponded with the active sites of Tyr from different sources were varied from 120 to 600 mV versus NHE [48]. The peak-to-peak separation Δ*E_p_* was 40 mV and about oneratio of cathodic to anodic current intensity at the scan rate of 0.1 V/s. The redox process of Tyr at the Nafion/Tyr/EGR-AuNPs-Ch/GCE gave roughly symmetric anodic and cathodic peaks at relative slow scan rates. When the scan rate increased, the redox potentials (*E_pa_* and *E_pc_*) of Tyr hardly shift. Meanwhile, the redox peak current increased linearly (inset, Figure 4): *I_pa_* = −3.7 × 10^−1^ − 1.4 × 10^−1^ *v*, *r* = 0.9996; *I_pc_* = 9.5 × 10^−1^ + 1.2 × 10^1^ *v*, *r* = 0.9996. The high electroactive area of EGR-AuNPs-Ch induces high capacitance [11] and high background currents varying proportionally with the scan rate. This indicated that the electron transfer process for Tyr at the Nafion/Tyr/EGR-AuNPs-Ch/GCE was a surface-confined mechanism in the abovementioned potential scope, manifesting the characteristics of the thin-layer surface-controlled electrochemical process.

The anodic and cathodic peak potentials were linearly dependent on the logarithm of the scan rates (*n*) with slopes of −2.3*RT*/*anF* and 2.3*RT*/(1−*a*)*nF*, respectively. Hence, the charge–transfer coefficient *a* was calculated to be 0.47. The heterogeneous electron transfer rate constant (*k_s_*) was further estimated according to the following equation [49]:

*log k_s_* = *a*log (1 − *a*) + (1 − *a*)*log a* − *log* (*RT*/*nFv*) − (1 − *a*)*aF*Δ*E_p_/*(2.3*RT*) (1)

where *a* is the charge transfer coefficient. *n* is the number of electrons transferred. *R*, *T*, and *F* symbols have their conventional meanings. Δ*E_p_* is the peak-to-peak potential separation.

The result was 0.66 s^−1^, which was higher than 0.032 s^−1^ for Tyr-AuNPs/boron-doped diamond (BDD) [50] and 0.030 s^−1^ for Tyr/AgE [22]. Thus, Nafion/Tyr/EGR-AuNPs-Ch/GCE can provide a favorable microenvironment for Tyr to undergo a facile electron transfer reaction due to the structure of EGR-AuNPs-Ch, which is to the benefit of effective immobilization of enzymes, proteins, and other bioactive substances. EGR greatly increased the specific surface area and shortens the distance between the active centers of Tyr and the electrode surface. Furthermore, interlayers offered more binding sites for the immobilization of Tyr.

### 3.3. Amperometric Response Towards CA

**Scheme 2:**
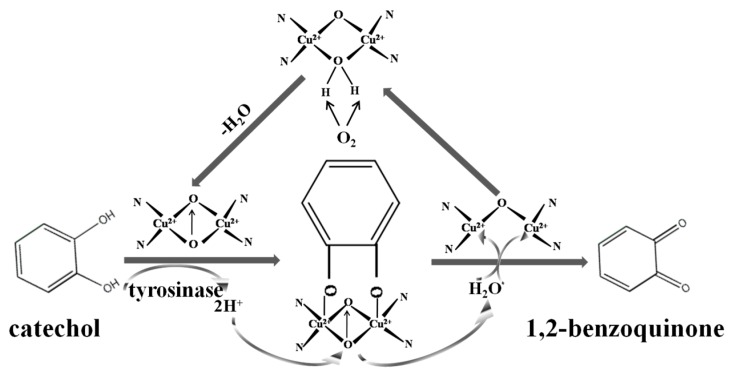
Schematics of the possible mechanism of catalytic oxidization of Tyr towards catechol with Nafion/Tyr/EGR-AuNPs-Ch/GCE.

The possible mechanism of electrocatalytic reduction of CA at the Tyr-based enzyme electrode can be expressed as follows (Scheme 2):

catechol + tyrosinase (O_2_) → *o*-quinone + H_2_O (2)

However, *o*-quinone is extremely unstable, an accompanying reaction followed [44]:

*o*-quinone + 2H^+^ + 2e^−^ → catechol (3)

**Scheme 3:**
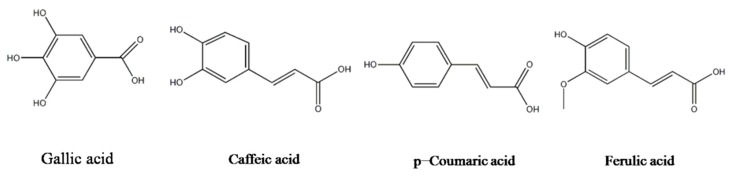
The structure ofgallic acid, caffeic acid, *p*-coumaric acid, and ferulic acid.

Similar structural compounds, such as gallic acid, caffeic acid, *p*-coumaric acid, and ferulic acid (as shown in Scheme 3) were also determined by the Nafion/Tyr/EGR-AuNPs-Ch/GCE. As derivatives of CA, the caffeic acid current response towards the same concentration was only one fifth to one third that of CA. The quite similar structure of caffeic acid made it easily catalyze by Tyr. The carboxyl group and double bond contributed to the current drop. Gallic acid showed slight current change. The third hydroxyl severely hindered the catalytic process of Tyr due to steric effects. There were no remarkable current change of *p*-coumaric acid and ferulic acid. Monophenols are hydroxylated to a variety of diphenols and then catalyzed by Tyr, followed by subsequent oxidation to quinines [51]. EGR can interact with the double bond of *p*-coumaric acid and ferulic acid by the π-π attraction, which blocks the monophenols’ hydroxylation, resulting in the difficulty of Tyr catalysis. The above results confirmed the mechanism of electrocatalytic reduction of CAwith Nafion/Tyr/EGR-AuNPs-Ch/GCE.

Chronoamperometry was also used for the investigation of electrocatalysis of CA with Nafion/Tyr/EGR-AuNPs-Ch/GCE. The effect of applied potential was investigated at different potentials ranging from 0.10 V to −0.30 V. At the applied potentials from 0.10 V to −0.10 V, the current increased and reached a peak plateau. From −0.10 V to −0.30 V, the response of the electrode gradually decreased. Moreover, the baseline current of the signal became unstable above −0.10 V. As a result, −0.10 V was finally chosen as the applied potential throughout all the amperometric measurements.

**Figure 5:**
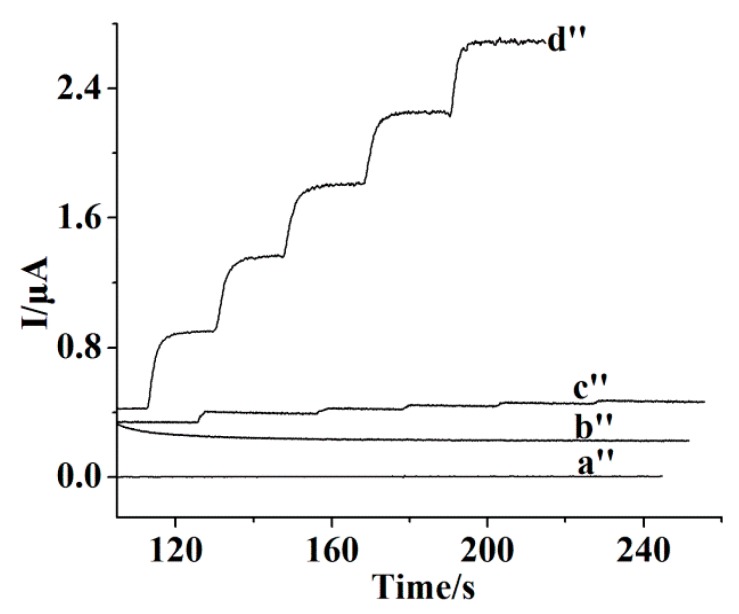
Amperometric responses ofthe bare GCE (a″), theAuNPs-Ch/GCE(b″), the EGR-AuNPs-Ch/GCE (c″) and Nafion/Tyr/EGR-AuNPs-Ch/GCE (d″) at −0.10 V (vs. SCE) upon successive addition of the same concentration of CA.

Figure 5 shows the amperometric curves performed to study the performance of the fabricated electrodes with the successive addition of CA. When CA was added into the stirring 0.1 M, pH 7.0 PBS, a quick response to the substrate occurred. The proposed Nafion/Tyr/EGR-AuNPs-Ch/GCE (d″) obtained the best amperometric response, as expected. Comparing with the bare GCE (a″), the AuNPs-Ch/GCE (b″) and the EGR-AuNPs-Ch/GCE (c″) upon the addition of 5 μM of CA into continuously-stirring PBS at −0.10 V, the current response of the Nafion/Tyr/EGR-AuNPs-Ch/GCE increased dramatically.

**Figure 6:**
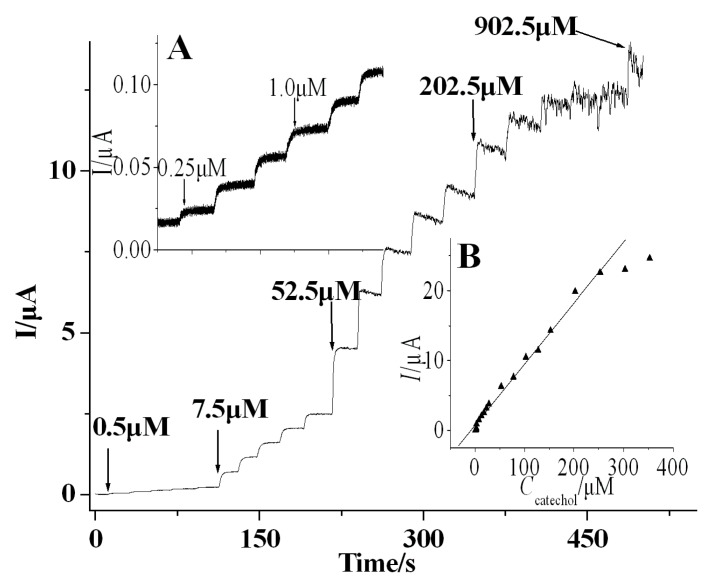
Amperometric responses of the Nafion/Tyr/EGR-AuNPs-Ch/GCE at −0.10 V (vs. SCE) upon successive addition of 0.5 μM, 7.5 μM, 52.5 μM, 202.5 μM, and 902.5 μM CA in PBS. Inset: (**A**) amperometricresponses of the Nafion/Tyr/EGR-AuNPs-Ch/GCE at 0.20 V (vs. SCE) upon successive addition of 0.25 μM and 1.0 μM CA in PBS; and (**B**) plot of peak current vs. CA concentration.

Figure 6 illustrated a typical amperometriccurve of the sensor based on Nafion/Tyr/EGR-AuNPs-Ch/GCE upon the addition of an aliquot concentration of CA into continuously-stirring 0.1 M, pH 7.0 PBS at optimal condition. Even with 0.25 μM of CA, as shown in Figure 6, inset A, obvious amperometric responses are shown. The 95% steady-state current can be obtained at 2–3 s, revealing the faster response of the sensor than that of previously-reported CA sensors [7,14]. The current response increased along with the CA concentration. The calibration curve at the biosensor showed linearity from 0.2 to 270 μM (Figure 6, inset B). The linear regression equation was obtained as *I_ss_* (μA) = 9.11 × 10^−2^*C* (μM) + 5.13 × 10^−1^ (*r* = 0.9962) with a detection limit of 0.1 μM (S/N = 3). The sensitivity obtained from the slope of the calibration curve is 122A/M·cm^−2^, which was much higher than the listed CA sensors in the literature [7]. The biosensor had a much better catalytic response towards CA than that of the agarose-guar gum-entrapped Tyr [10] and PANI-polyphenol oxidase (PPO) film [14].

According to Michaelis-Menten kinetic mechanism, the apparent Michaelis-Menten constant $K_{M}^{app}$ can be obtained from the electrochemical version of the Lineweaver-Burk equation [52,53]:

(4) $$1/I_{ss}=1/I_{max}+K_{M}^{app}/(I_{max}C)$$

Here, *I_ss_* is the diffusion-limiting current after the addition of the substrate. *C* is the bulk concentration of the substrate, and *I_max_* is the maximum current measured under the saturated substrate conditions. The value of $K_{M}^{app}$ and *I_max_* can be obtained by the slope and the intercept of the plot of the reciprocals of the steady-state current versus CA concentration. The apparent Michaelis-Menten constant $K_{M}^{app}$ value was calculated to be 109 μM, which was much lower than some previous reports [14,54]. The $K_{M}^{app}$ value reflects the affinity of the enzyme for the substrate: the smaller the $K_{M}^{app}$ value, the greater the affinity [55]. Thus, the lower value of $K_{M}^{app}$ indicates that the immobilized Tyr exhibited stronger affinity to CA than that of the free Tyr [56]. Tyr can also retain higher activity in Nafion/Tyr/EGR-AuNPs-Ch/GCE. This methodology provides grapheme-nanomaterial composite films with low cost, no chemical use, massive parallelism, and controllability of the nanomaterial composition.

### 3.4. Repeatability and Stability of CA Biosensor

The stability and repeatability of the biosensor were studied. The relative standard deviation (RSD) was 2.4% for eight successive measurements of 10 μM CA in PBS, showing that the proposed biosensor possessed good repeatability. The cyclic voltammetric responses of the modified biosensor in PBS containing 10 μM CA showed no obvious change after 25 cycles, and then it decreased slowly with the increase of the cycle, indicating that the biosensor was stable. The storage stability the biosensor was further investigated. The peak currents of the Nafion/Tyr/EGR-AuNPs-Ch/GCE was measured using the same electrode and it retained above 95% of its initial response stored at 4 °C after three weeks. These results displayed that the biosensor based on the Nafion/Tyr/EGR-AuNPs-Ch/GCE had good stability.

## 4. Conclusions

In the present work, GR was successfully prepared in situ on choline-functionalized gold nanoparticle-modified GCE. Based on the EGR-AuNPs-Ch/GCE platform, the CA biosensor exhibited a variety of good electrochemical characteristics, including a low detection limit, high catalytic ability, wide linearity, and a larger electron transfer rate constant of 0.66 s^−1^. These advantages should be attributed to the following: (1) AuNPs-Ch can be efficiently immobilized on the bare GCE, and interlayer EGR provides more attachment sites for Tyr immobilization; (2) π-π electron transfer between EGR, plays an important role in facilitating the electron transfer between Tyr and the electrode surface; and (3) synergistic effects of AuNPs-Ch and EGR exhibited the signal amplification of nanosized materials.
